# Active Crossfire Between Cyanobacteria and Cyanophages in Phototrophic Mat Communities Within Hot Springs

**DOI:** 10.3389/fmicb.2018.02039

**Published:** 2018-09-03

**Authors:** Sergio Guajardo-Leiva, Carlos Pedrós-Alió, Oscar Salgado, Fabián Pinto, Beatriz Díez

**Affiliations:** ^1^Department of Molecular Genetics and Microbiology, Pontificia Universidad Católica de Chile, Santiago, Chile; ^2^Programa de Biología de Sistemas, Centro Nacional de Biotecnología – Consejo Superior de Investigaciones Científicas, Madrid, Spain; ^3^Center for Climate and Resilience Research, Santiago, Chile

**Keywords:** hot-springs, cyanophages, phototrophic microbial mat, CRISPR, thermophilic cyanobacteria

## Abstract

Cyanophages are viruses with a wide distribution in aquatic ecosystems, that specifically infect Cyanobacteria. These viruses can be readily isolated from marine and fresh waters environments; however, their presence in cosmopolitan thermophilic phototrophic mats remains largely unknown. This study investigates the morphological diversity (TEM), taxonomic composition (metagenomics), and active infectivity (metatranscriptomics) of viral communities over a thermal gradient in hot spring phototrophic mats from Northern Patagonia (Chile). The mats were dominated (up to 53%) by cosmopolitan thermophilic filamentous true-branching cyanobacteria from the genus *Mastigocladus*, the associated viral community was predominantly composed of Caudovirales (70%), with most of the active infections driven by cyanophages (up to 90% of Caudovirales transcripts). Metagenomic assembly lead to the first full genome description of a T7-like Thermophilic Cyanophage recovered from a hot spring (Porcelana Hot Spring, Chile), with a temperature of 58°C (TC-CHP58). This could potentially represent a world-wide thermophilic lineage of podoviruses that infect cyanobacteria. In the hot spring, TC-CHP58 was active over a temperature gradient from 48 to 66°C, showing a high population variability represented by 1979 single nucleotide variants (SNVs). TC-CHP58 was associated to the *Mastigocladus* spp. by CRISPR spacers. Marked differences in metagenomic CRISPR *loci* number and spacers diversity, as well as SNVs, in the TC-CHP58 proto-spacers at different temperatures, reinforce the theory of co-evolution between natural virus populations and cyanobacterial hosts. Considering the importance of cyanobacteria in hot spring biogeochemical cycles, the description of this new cyanopodovirus lineage may have global implications for the functioning of these extreme ecosystems.

## Introduction

Hot springs host microbial communities dominated by a limited variety of microorganisms that form well-defined mats (Uldahl and Peng, 2013; Inskeep et al., 2013). Frequently, the uppermost layer of the mat is composed of photoautotrophs; such as oxygenic phototrophic cyanobacteria, including the unicellular cyanobacterium *Synechococcus* spp. (Steunou et al., 2006, 2008; Bhaya et al., 2007; Klatt et al., 2011), the filamentous non-heterocystous *Oscillatoria* spp., the filamentous heterocystous *Mastigocladus* spp. (Stewart, 1970; Miller et al., 2006; Mackenzie et al., 2013; Alcamán et al., 2015), as well as filamentous anoxygenic phototrophs (FAPs), such as *Roseiflexus* sp. and *Chloroflexus* sp. (Van der Meer et al., 2010; Klatt et al., 2011; Liu et al., 2011). These primary producers interact with heterotrophic prokaryotes through element and energy cycling (Klatt et al., 2013). Heterocystous cyanobacteria are a key component in hot springs, since these systems are commonly N-limited due to the rapid assimilation and turnover of inorganic nitrogen forms (Alcamán et al., 2015; Lin et al., 2015). Thus, N_2_-fixation by cyanobacteria is identified to be a key biological process in neutral hot spring microbial mats (Alcamán et al., 2015).

These simplified but highly cooperative communities have been historically used as models for understanding the composition, structure, and function of microbial consortia (Klatt et al., 2011; Inskeep et al., 2013). The role of a variety of abiotic factors, such as pH, sulfide concentration, and temperature, in determining microbial assemblages and life cycles in these ecosystems have been investigated (Cole et al., 2013; Inskeep et al., 2013). However, there is a lack of investigation into biotic factors, such as viruses, on thermophilic photoautotrophic mats, with existing studies only reporting short or partial viral sequences (Heidelberg et al., 2009; Davison et al., 2016). Currently, viral communities from thermal mats have been characterized through indirect approaches, indicating the hypothetical presence of viruses (Heidelberg et al., 2009; Davison et al., 2016). Heidelberg et al. (2009) used CRISPR spacer sequences extracted from the genomes of two thermophilic *Synechococcus* isolates, from a phototrophic mat in Octopus Spring. Subsequently, they searched for viral contigs from previously published water metaviromes from the Octopus and Bear Paw Springs in Yellowstone National Park (United States) (Schoenfeld et al., 2008). Furthermore, Davison et al. used CRISPR spacers and nucleotide motive frequencies to link viral contigs to known hosts using a metavirome obtained by Multiple Displacement Amplification (MDA) of VLPs from a mat in Octopus Spring (Davison et al., 2016), as well as reference genomes from dominant species (*Synechococcus* sp., *Roseiflexus* sp., and *Chloroflexus* sp.) previously described in the same microbial mat. A key finding from these studies was the link between viruses and their hosts, indicating their co-evolution and an effective “arms race” within hot spring phototrophic mats.

Unlike thermophilic mat studies, most viral investigation carried out in hot springs occur within the source waters (Rachel et al., 2002; Yu et al., 2006; Schoenfeld et al., 2008; Bolduc et al., 2012, 2015; Zablocki et al., 2017). In these waters, virus abundances range between 10^4^ and 10^9^ virus like particles (VLPs) mL^-1^ (Breitbart et al., 2004; Schoenfeld et al., 2008; Redder et al., 2009). They play an important role in both the structuring of host populations and as drivers of organic and inorganic nutrient recycling (Breitbart et al., 2004). The majority of the viruses were dsDNA, with new and complex viral morphotypes, distinct to the typical head and tail morphologies (Rachel et al., 2002; Prangishvili and Garrett, 2004; Schoenfeld et al., 2008; Redder et al., 2009; Pawlowski et al., 2014). Furthermore, the few metaviromes obtained in thermal waters indicate that natural thermophilic virus communities differ from those obtained in culture, given that there was only a 20–50% similarity between the sequences obtained and those in the databases (Pride and Schoenfeld, 2008; Schoenfeld et al., 2008; Diemer and Stedman, 2012; Bolduc et al., 2015). Thus far, the genomes that have been isolated and sequenced from thermophilic viruses (57 genomes, of which 37 infected archaea and 20 infected Bacteria) generally yielded few significant matches to sequences in public databases (Uldahl and Peng, 2013). More recently, a water metaviromic study from Brandvlei hot spring (BHS), South Africa (Zablocki et al., 2017) reported the presence of two partial genomes (10 kb and 27 kb), the first related to Podoviridae and the second to lambda-like Siphoviridae families. Both Caudovirales genomes did not have a confirmed host, but the presence of green microbial mat-patches around the contours of the hot spring, implied that filamentous Cyanobacteria and unclassified *Gemmata* species were the potential hosts, respectively. The last, based on the proximity of some viral predicted proteins with bacteria from well characterized microbial mats present in a nearby hot spring (Tekere et al., 2011; Jonker et al., 2013).

Given the lack of knowledge of viral communities within hot spring phototrophic microbial mats, the present study used the mats of Porcelana hot spring (Northern Patagonia, Chile), as a pH neutral model, to better understand the associated thermophilic viral communities within these mats. This pristine spring is covered by microbial mats that grow along a thermal gradient between 70 and 46°C, dominated by bacterial phototrophs, such as filamentous cyanobacteria from the genus *Mastigocladus* (Mackenzie et al., 2013; Alcamán et al., 2015). This is the dominant and most active cyanobacterial genus in the Porcelana mat environment, carrying out important biological processes such as carbon- and N_2_-fixation (Alcamán et al., 2015, 2017). Thus, this study proposes that the mats in Porcelana hot spring are dominated by viral communities of the Order Caudovirales, which is able to infect Cyanobacteria, preferably *Mastigocladus* spp.

The viral diversity in Porcelana was determined through the detection of viral signals in microbial mat omics data, and by TEM along the thermal gradient. The results demonstrate that the viral community was dominated by Caudovirales, which actively infect Cyanobacteria. Furthermore, the first complete genome description of a thermophilic cyanobacterial T7-like podovirus, Thermophilic Cyanophage Chile Porcelana 58°C (from now on TC-CHP58) is realized. The host is the dominant phototroph *Mastigocladus* spp, based on CRISPR spacers. Finally, the presence of different populations of this new podovirus are identified through single nucleotide variants (SNVs) analyses, and the co-evolution of *Mastigocladus* spp. and particular populations of TC-CHP58 at different temperatures is described through association of specific SNVs to different CRISPR spacers.

## Materials and Methods

### Sampling Site

Porcelana hot spring is located in Chilean Patagonia (42° 27′ 29.1^′′^S – 72° 27′ 39.3^′′^W). It has a neutral pH range between 7.1 and 6.8 and temperatures ranging from 70 to 46°C, when sampled on March 2013. Phototrophic microbial mats growing at 66, 58, and 48°C were sampled using a cork borer of 7 mm diameter. Cores of 1 cm thick were collected in triplicate at noon (12:00 PM), transported in liquid nitrogen and kept at -80°C until DNA and RNA extraction.

### Transmission Electron Microscopy

Five liters of interstitial fluid was squeezed using 150 μm sterilized polyester net SEFAR PET 1000 (Sefar, Heiden, Switzerland) and filtered through 0.8 μm pore-size polycarbonate filters (Isopore ATTP, 47 mm diameter, Millipore, Millford, MA, United States) and 0.2 μm pore-size (Isopore GTTP, 47 mm diameter, Millipore) using a Swinex filter holder (Millipore). Particles in the 0.2 μm filtrate were concentrated to a final volume of approximately 35 ml using a tangential-flow filtration cartridge (Vivaflow 200, 30 kDa pore size, Vivascience, Lincoln, United Kingdom). Viral concentrates (15 μL) were spotted onto Carbon Type-B, 200 mesh, Copper microscopy grids (Ted Pella, Redding, California, United States), stained with 1% uranyl acetate and imaged on an FEI Tecnai T12 electron microscope at 80 kV (FEI Corporate, Hillsboro, OR, United States) with attached Megaview G2 CCD camera (Olympus SIS, Münster, Germany). Imaging analysis was done at the Advanced Microscopy Unit, School of Biological Sciences at Pontificia Universidad Católica de Chile (Santiago, Chile).

### Nucleic Acid Extractions and High Throughput Sequencing

Nucleic acids (DNA and RNA) were extracted as previously described (Alcamán et al., 2015). For RNA, Trizol (Invitrogen, Carlsbad, CA, United States) was added to the mat sample, and homogenized by bead beating, two pulses of 20 s. Quality and quantity of the extracted nucleic acids were checked and kept at -80°C.

Samples were sequenced by Illumina Hi-seq technology (Research and Testing Laboratory, Texas, United States). Briefly, for metagenomes, DNA was fragmented using NEBNext dsFragmentase (New England Biolabs, Ipswich, MA, United States), followed by DNA clean up using column purification, and a NEBUltra DNA Library Prep Kit for Illumina (New England Biolabs, Ipswich, MA, United States) was used for library construction.

For metatranscriptomes, DNase treated total RNA was cleaned up of rRNA by a Ribo-Zero rRNA Removal Kit Bacteria (Illumina, San Diego, CA, United States), followed by purification using an Agencourt RNAClean XP Kit (Beckman Coulter, Indianapolis, IN, United States), and a NEXTflex^TM^ Illumina Small RNA Sequencing Kit v3 (Bio Scientific, Austin, TX, United States) was used for library construction.

For quality filtering, the following filters were applied using Cutadapt (Martin, 2011), leaving only mappable sequences longer than 30 bp (-m 30), with a 3′ end trimming for bases with a quality below 28 (-q 28), a hard clipping of the first five leftmost bases (-u 5), and finally a perfect match of at least 10 bp (-O 10) against the standard Illumina adaptor. Finally, the removal of sequences representing simple repetitions that are usually due to sequencing errors was applied using PRINSEQ (Schmieder and Edwards, 2011) DUST threshold 7 (-lc_method dust, -lc_threshold 7). Details of the number of sequences obtained are shown in **Supplementary Table S1**.

### Identification of rRNA-Like Sequences and Viral Mining From Metagenomes and Metatranscriptomes

Metagenomic Illumina TAGs (miTAGs) (Logares et al., 2014) that are small subunit (SSU) 16S and 18S rRNA gene sequences in the metagenomes were identified and annotated using the Ribopicker tool (Schmieder et al., 2012) with the Silva 123 SSU database (Quast et al., 2013).

For viral mining, bacterial, archaeal and eukaryotic sequences were removed through end-to-end mapping, allowing a 5% of mismatch (-N 1 -L 20) against the NCBI non-redundant (NR) database (Nov-2015) using bowtie2 (Langmead and Salzberg, 2012). Viral sequences were then recruited against modified NCBI RefSeq (Release 75) viral proteins, where only amino acid sequences from viruses that do not infect animals (NAV) were considered to build the database, using the UBLAST algorithm (-strand both -accel 0.9) through the USEARCH sequence analysis tool (Edgar, 2010). Recruitment was made for sequences with over 65% of coverage and an E-value < 1 × 10^-3^ (-query_cov 0.65 -evalue 1e-3). For taxonomic assignment, recruited sequences were aligned against the NAV database using BLASTX (Camacho et al., 2009) and parsed using the lowest common ancestor algorithm trough MEGAN 6 (Huson et al., 2016) (LCA score = 30). The latter displays a graphical representation of abundance for each taxonomic group identified at the family and species levels. Species classification of viral reads, was used to infer the phyla of the putative hosts based on viral RefSeq host information or through a manual search of the publication associated with each viral genome.

To extract putative viral genomes, all metagenomes (48, 58, and 66°C) were assembled using De Bruijn graphs as implemented in the Spades assembler (Bankevich et al., 2012), followed by gene prediction using Prodigal software (Hyatt et al., 2010) and the recovery of circular contigs over 5 kb using a Python script (Crits-Christoph et al., 2016). Only sequences over 5 kb were used in the subsequent analysis because all dsDNA viruses in the databases have genomes over that size. A homology search of the viral predicted proteins by Prokka (Seemann, 2014) was done using BLASTX against the NAV protein database and NCBI nr as described before. Additionally, all contigs over 5 kb were analyzed using VirSorter (Roux et al., 2015a) against the virome database option.

To quantify the abundance and activity of the retrieved viral genome, reads recruitment from each metagenome and metatranscriptome was performed using BWA-MEM (-M), resulting SAM file was parsed by BBmap pileup script (Bushnell B.) ^1^.

### Phylogenetic Analysis

The protein inferred sequences of DNA polymerase and major capsid were aligned by Muscle (Edgar, 2004) and MAFFT (Katoh et al., 2002), respectively, using the amino acid substitution model determined by ProtTest 3 (Blosum62+G+F) (Darriba et al., 2011) and modelFinder (LG+F+G4), respectively. The Bayesian Markov chain Monte Carlo method was implemented with MrBayes 3.6 (Ronquist et al., 2012) and MCMC results were summarized with Tracer 1.6^2^. MrBayes was run using two independent runs, four chains, 1,500,000 generations and a sampling frequency of 100 with a burn-in value of 33% until the standard deviations of split frequencies remained below 0.01.

The maximum likelihood method was implemented with IQtree (-bb 10000 -nm 10000 -bcor 1 -numstop 1000) (Trifinopoulos et al., 2016) using 100 standard bootstrap and 10,000 ultrafast bootstrap to evaluate branch supports. The details of the sequences used for phylogenetic analyses are listed in **Supplementary Table S2**.

### CRISPR/Cas Virotopes

Assemblies for each temperature, were taxonomically grouped (bins) using the Expectation–Maximization (EM) algorithm implemented in MaxBin 2.0 (Wu et al., 2016). In order to asses the completeness and contamination of each bin, CheckM (Parks et al., 2015) analyses were performed. Finally, the closest genome of each bin was searched using the Tetra Correlation Search (TCS) analysis implemented in Jspecies tool (Richter et al., 2016) with selection criteria of Z score greater than 0.999 and ANI over 95% (Konstantinidis et al., 2017).

CRISPR/Cas *loci* were identified in contigs assigned to *Mastigocladus* spp. from 48, 58, and 66°C assembled metagenomes using CRISPRFinder tool (Grissa et al., 2007). To quantify the activity of the CRISPR *loci*, reads recruitment from metatranscriptomes for the same temperatures was performed using BWA-MEM (-M), and the resulting SAM file was parsed by BBmap pileup script (Bushnell B.) (see footnote 1) and normalized by total number of reads and length of each *loci*.

Spacers from CRISPR containing contigs were mapped to viral contigs using bowtie2 (Langmead and Salzberg, 2012) parameters (-end-to-end -very sensitive -N 1). Mapped spacers were manually annotated to the viral predicted proteins in viral contig.

### Single Nucleotide Variants (SNVs)

To call variants occurring in TC-CHP58 populations at the three different metagenome temperatures, LoFreq method (Wilm et al., 2012) was used. SNVs frequencies were quantified in ORFs from TC-CHP58 genome using Bedtools suite (Quinlan and Hall, 2010). The alleles of SNVs present in proto-spacers were visualized in IGV tools for each virotope at each temperature.

## Results

### Morphological and Genetic Composition of VLPs

**FIGURE 1:**
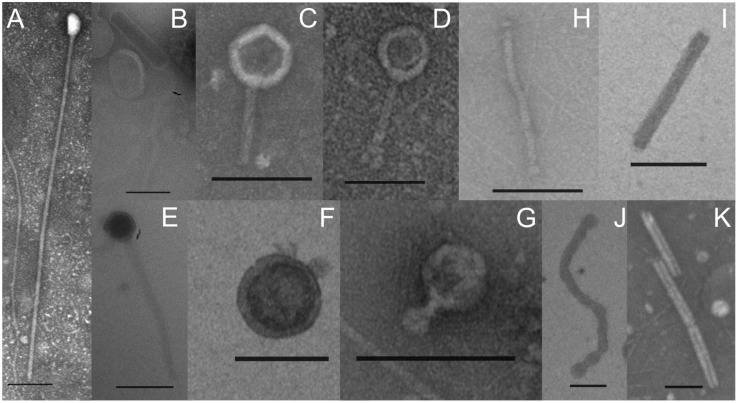
Transmission electronic micrographs of VLPs obtained from the interstitial fluid of phototrophic microbial mats growing between 62°C and 42°C in Porcelana hot spring. Scale bar: 100 nm. **(A–G)** Caudovirus-like particles belonging to Myoviridae, Podoviridae, and Siphoviridae families. **(H–K)** Filamentous and rod shaped VLPs that could be associated with Lipothrixviridae and Clavaviridae families.

**FIGURE 2:**
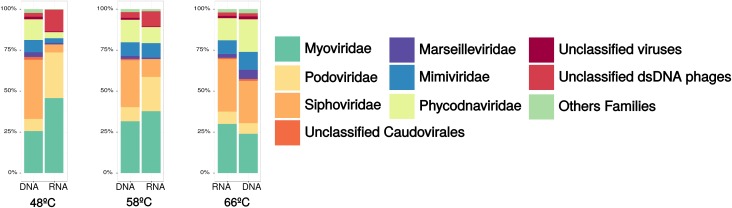
Relative abundances of viral Families in microbial mats from Porcelana hot spring; standardized by the total number metagenomic reads (DNA), and metatranscriptome (RNA) from each temperature samples.

Transmission electron microscopy (TEM) was applied to identify the VLPs present in the interstitial fluid from microbial mats in Porcelana hot spring. Caudovirus-like particles belonging to Myoviridae, Podoviridae and Siphoviridae families, typically infecting bacteria (**Figures 1A–G**) were identified. Additionally, filamentous and rod shaped VLPs were detected, that could be associated with Lipothrixviridae and Clavaviridae families, usually infecting archaea (**Figures 1H–K**). Viral read counts ranged between 0.47 and 0.78% of the total metagenome reads, and between 0.35 and 3.71% in the metatranscriptomes (**Supplementary Table S1**). At all temperatures, viral metagenomic sequences (**Figure 2**) revealed the dominance of the Order Caudovirales, followed by the Order Megavirales, with ∼70% and ∼23% of the total viral reads, respectively. Metatranscriptomic analysis results (**Figure 2**) showed a slightly different pattern, with a reduction in Caudovirales with increasing temperature (from ∼78% at 48°C to ∼57% at 66°C), whereas Megavirales did the opposite (from ∼7% at 48°C to ∼36% at 66°C).

In the metagenomes, Siphoviridae was the most abundant family of Caudovirales, with maximum abundance at 48°C. Myoviridae members were also well represented with a maximum of ∼31% at 58°C and a minimum (∼25%) at 48°C. Meanwhile, Podoviridae accounted for just ∼8% at all temperatures (**Figure 2**). In metatranscriptomes, Siphoviridae increased sixfold with temperature, while Podoviridae and Myoviridae decreased with temperature (fivefold and twofold, respectively).

The Megavirales order was also present, however, at a lower abundance compared to Caudovirales. Megavirales were represented by Phycodnaviridae (∼13%), Mimiviridae (∼8%), and Marseilleviridae (∼2%) families, remaining constant through all temperatures. Metatranscriptomics showed an increase in abundance of these three virus families with temperature.

### Caudovirales Host Assignments

**FIGURE 3:**
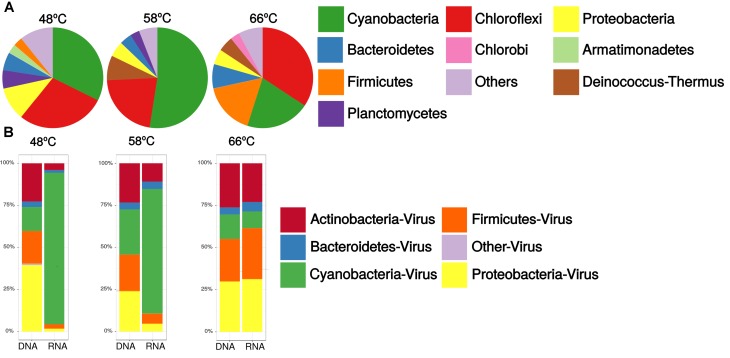
Relative abundances of **(A)** Bacterial community, to Phylum level, in the microbial mats obtained from 16S miTAGs, standardized by the total number of metagenomic reads (DNA) for each temperature sample, and **(B)** Caudovirales community at the host Phylum level, obtained from shotgun sequences in metagenomes (DNA) and metatranscriptomes (RNA), standardized by the total number of reads from each temperature sample.

Porcelana mat communities based on miTAGs were dominated by bacteria (∼96%), with low abundances of eukarya (∼3%) and archaea (∼1%) (**Supplementary Table S1**). At the phylum level (**Figure 3A**), bacterial communities were mostly composed of Cyanobacteria oxygenic phototrophs (33, 53, and 21% of total rRNA SSU sequences at 48, 58, and 66°C, respectively) and Chloroflexi anoxygenic phototrophs (higher than Cyanobacteria only at 66°C, with 35% of total rRNA SSU sequences). Other representative members of the community were Proteobacteria (5–11%), Deinococcus–Thermus (2–7%), Firmicutes (1–17%), and Bacteroidetes (4–8%) (**Figure 3A**).

The host assignment, based on taxonomy from viral reads of the most representative Caudovirales (**Figure 3B**), showed that viruses putatively infected members of the bacterial phyla Proteobacteria, Cyanobacteria, Actinobacteria, and Firmicutes. Metagenomic data showed that increases in temperature led to an increase in viruses from Actinobacteria and Firmicutes. Additionally, an increase in Cyanobacteria viruses was observed at 58°C. Viruses from Proteobacteria, Actinobacteria, and Firmicutes were represented by the three Caudovirales families, while viruses from Cyanobacteria were represented by Podoviridae and Myoviridae families only (**Supplementary Table S3**), where cyanopodovirus and cyanomyovirus reads increase from 31 to 50% at 48°C and from 30 to 45% at 58°C, then decrease to 23 to 28% at 66°C, respectively.

Metatranscriptomic sequences from Caudovirales potentially infecting Cyanobacteria, were predominant at 48°C and 58°C, with ∼90% and ∼74% of the total viral sequences, respectively. However, cyanophage transcripts abruptly decrease at 66°C. Cyanophages were exclusively related to the Myoviridae and Podoviridae families (**Supplementary Table S3**). Reads associated with cyanopodoviruses and cyanomyoviruses gradually decreased with temperature; between 48 and 58°C, virus reads declined from 95% and 96% to 84% and 89%, respectively. On the other hand, at 66°C a more severe decline was observed, to 15% and 20%, respectively. Conversely, with the reduced representation of Cyanobacteria at 66°C, other caudovirales transcripts increased, including those that infect Proteobacteria (∼31%), Firmicutes (∼30%), and Actinobacteria (∼23%).

### Thermophilic Cyanophage Genome Recovery

**FIGURE 4:**
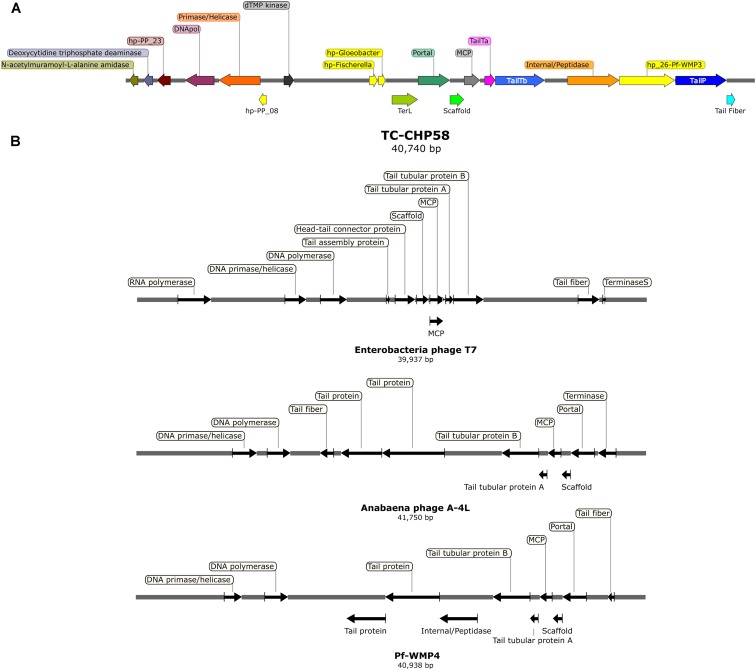
**(A)** Genomic organization of the thermophilic cyanophage CHP58 (TC-CHP58). Arrows indicate the size, position, and orientation of annotated ORFs, with predicted functions or homologs (e.g., DNApol, DNA polymerase; TailY a/b, tail tubular protein a/b; MCP, major capsid protein; TerL, large terminase subunit; TailP, Tail protein; hp-PP_08/23, homologous to hypothetical proteins 08/23 from cyanophage PP; hp-*Fischerella/Gloeobacter*, homologous to hypothetical proteins in *Fischerella* sp. PCC 9605/*Gloeobacter kilaueensis*). **(B)** Genomic organization of Enterobacteria phage T7, Anabaena phage A4L and Pf-WMP4. Arrows indicate the size, position, and orientation of viral core ORFs.

**FIGURE 5:**
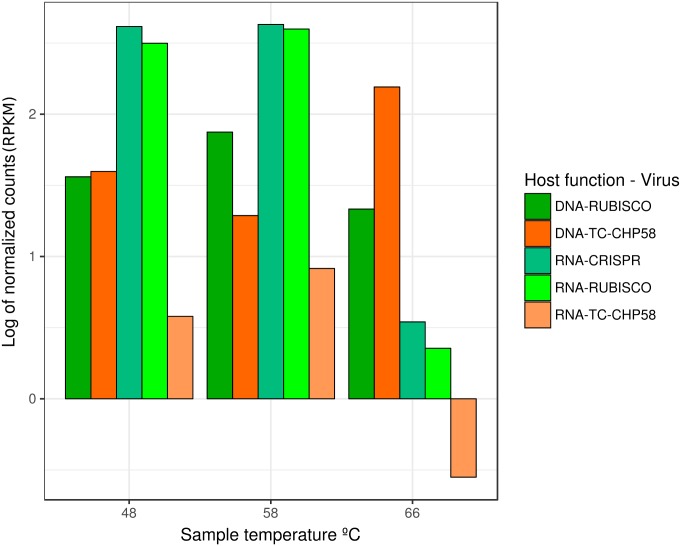
Relative abundance and transcriptomic expression of *Mastigocladus* spp. CRISPR systems and TC-CHP58. Abundance and expression of *Mastigocladus* RUBISCO was used as reference of the cyanobacterial presence and metabolic activity. Only specific CRISPR *loci* with proto-spacers in TC-CHP58 were fully quantified for each temperature. For improved visualization, counts are represented as Log of reads per kilobase million (RPKM).

The metagenome assembly recovered 3,912; 2,697; and 2,758 contigs, at 48°C, 58°C, and 66°C, respectively. A script search (Crits-Christoph et al., 2016) resulted in 11 circular contigs, possibly indicating complete genomes. Subsequent BLASTP analysis (Camacho et al., 2009) of predicted proteins indicated that only one circular contig had viral hallmark genes, meanwhile nine contigs had genes associated with bacterial mobile genetic elements and one contig remain completely unknown. These hallmark genes are shared by many viruses but are absents from cellular genomes (Koonin et al., 2006). VirSorter tool analysis (Roux et al., 2015a) confirmed these results, obtaining the same complete putative viral contig from the 58°C assembly, 40,740 bp long and 43.9% of GC content. This contig, TC-CHP58 (**Figure 4A**), was associated with a Cyanobacterial host. TC-CHP58 was present (reads recruitment) over all temperatures in Porcelana hot spring (**Figure 5** and **Supplementary Figure S1**). At 66°C, TC-CHP58 was sevenfold more abundant than their putative host (measured as *Mastigocladus* RUBISCO gene abundance); at 48°C, the virus-host ratio was 1:1, and at 58°C the host was fourfold more abundant than TC-CHP58. Metatrancriptomic reads also show that TC-CHP58 was active over all temperatures (**Figure 5** and **Supplementary Figure S2**), but with lower transcription levels than the putative host (measured as *Mastigocladus* RUBISCO gene activity), ranging between 80- and 8-fold lower (**Supplementary Table S4**). TC-CHP58 viral DNA:RNA ratio indicated similar proportions (2.4) at 58°C, least similar (552.9) at 66°C; while at 48°C the ratio was 10.4 (**Supplementary Table S4**).

### Genomic Features and Organization of TC-CHP58

Complete protein prediction and annotation of TC-CHP58 using Prokka (Seemann, 2014) and BLASTP revealed 39 putative ORFs, 10 of which were viral core proteins (i.e., capsid and tail-related proteins, DNA polymerase, Terminase, etc.), 22 had no significant similarities in NCBI nr database, and 4 were present in the database but with unknown function (**Table 1**).

**Table 1:** Blastp analysis of predicted CDS from TC-CHP58 of known function against NCBI RefSeq (Release 75) and NR databases.

Query sequence ID	Subject sequence ID	Identity %	E-value	Bit Score
TC-CHP58_sequence	KF598865.1| [Cyanophage PP]	93%	0.2	54.7
TC-CHP58_CDS1	YP_009042789.1| DNA polymerase [Anabaena phage A-4L]	29.03	4.00E-60	223
TC-CHP58_CDS3	YP_009042786.1| DNA primase/helicase [Anabaena phage A-4L]	25.21	2.00E-28	131
TC-CHP58_CDS4	YP_008766966.1| hypothetical protein PP_08 [Cyanophage PP]	29.7	0.0006	47
TC-CHP58_CDS7	WP_026824764.1| dTMP kinase [*Exiguobacterium marinum*]	30.61	4.00E-22	99.4
TC-CHP58_CDS13	WP_026731322.1| hypothetical protein [*Fischerella* sp. PCC 9605]	34.55	7.00E-12	69.3
TC-CHP58_CDS14	WP_023172199.1| hypothetical protein [*Gloeobacter kilaueensis*]	42.31	4E-05	48.1
TC-CHP58_CDS15	YP_008766995.1| terminase [Cyanophage PP]	44.39	2.00E-150	456
TC-CHP58_CDS16	YP_001285799.1| portal protein [Phormidium phage Pf-WMP3]	42.96	0	551
TC-CHP58_CDS17	YP_009042804.1| scaffold protein [Anabaena phage A-4L]	30.69	1E-07	60.8
TC-CHP58_CDS18	YP_008766991.1| capsid protein [Cyanophage PP]	48.14	4.00E-109	335
TC-CHP58_CDS20	YP_009042802.1| tail tubular protein A [Anabaena phage A-4L]	29.52	3.00E-28	116
TC-CHP58_CDS21	YP_001285795.1| tail tubular protein B [Phormidium phage Pf-WMP3]	36.49	0	630
TC-CHP58_CDS24	YP_009042798.1| internal protein [Anabaena phage A-4L]	29.86	5.00E-41	174
TC-CHP58_CDS25	YP_001285791.1| PfWMP3_26 [Phormidium phage Pf-WMP3]	28.26	5.00E-23	117
TC-CHP58_CDS26	YP_009042796.1| tail protein [Anabaena phage A-4L]	24.8	4.00E-89	322
TC-CHP58_CDS32	WP_038085449.1|*N*-acetylmuramoyl-L-alanine amidase [*Tolypothrix bouteillei*]	46.29	3.00E-46	160
TC-CHP58_CDS35	WP_043587103.1| deoxycytidine triphosphate deaminase [*Diplosphaera colitermitum*]	44.9	2.00E-48	167
TC-CHP58_CDS37	YP_008766981.1| hypothetical protein PP_23 [Cyanophage PP]	29.92	1.00E-21	101

Blast analysis of the viral genes in TC-CHP58, revealed 25 to 48% identity (amino acidic level) with proteins from Cyanophage PP, PF-WMP3 and Anabaena phage A-4L, that infect freshwater filamentous Cyanobacteria such as *Phormidium, Plectonema*, and *Anabaena* (**Table 1**). At the nucleotide level, there was almost no similarity to any known sequence except for a short segment of 40 nucleotides, which showed 93% similarity to a Portal protein gene sequence of *Plectonema* and P*hormidium* cyanopodoviruses (Cyanophage PP; NC_022751 and PF-WMP3; NC_009551).

Gene prediction by Prodigal indicated that the TC-CHP58 genome might be structured into two clusters, based on the transcriptional direction and putative gene functions (**Figure 4A**). The predicted ORFs (**Table 1**) in the sense strand encode proteins involved in DNA replication and modification, such as DNA polymerase and DNA primase/helicase. Conversely, the ORFs in the antisense strand (**Table 1**) encode proteins necessary for virion assembly, such as major capsid protein (MCP), tail fiber proteins, internal protein/peptidase, tail tubular proteins, scaffold protein, and portal protein. Moreover, two ORFs in the antisense strand had the best hits to the cyanobacterial hypothetical proteins found in the filamentous cyanobacterium *Fischerella* (WP_026731322. 1) and the unicellular *Gloeobacter* (WP_023172199.1).

Additionally, VIRFAM (Lopes et al., 2014) was used to classify TC-CHP58 according to their neck organization (**Supplementary Figure S3**), being assigned to the Podoviridae Type 3 category with neck structural organization similar to the Enterobacteria phage P22 (Lopes et al., 2014). Hierarchical clustering of neck proteins grouped TC-CHP58 together with the freshwater cyanophages Pf-WMP3 and Pf-WMP4, separating them from marine cyanophages such as P60 and Syn5.

Even when a large number of viral reads were assigned to cyanophages of Myoviridae family, it was not possible to recover any genome of this type. Most of the Myoviridae related contigs only had non-structural genes or hypothetical proteins of unknown function which align with proteins of known cyanomyoviruses. Here, the absence of hallmark genes from Cyanobacteria related viruses makes their accurate classification as cyanomyoviruses impossible.

### Phylogenetic Analysis of Phage TC-CHP58

**FIGURE 6:**
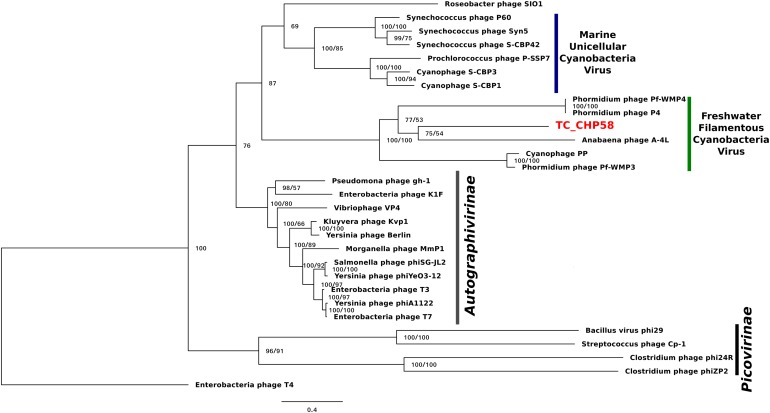
Bayesian inference phylogenetic reconstruction of DNA polymerase I protein of TC-CHP58. Numbers indicate Bayesian posterior probabilities as percentage/ultra-fast bootstrap values. Only UFBoot values over 80 and Bayesian PP over 50 are shown. The sequence characterized in the present study is reported in bold letters. Scale bar: 0.4 amino acid substitutions per site.

To investigate the relationship of the phage TC-CHP58 within the Podoviridae family, the DNApol gene was selected for comparison, using published viral genomes. The analysis included representatives of Picovirinae and Autographivirinae subfamilies, plus all the available DNApol genes from known freshwater podoviruses (Pf-WMP3, PP, Pf-WMP4 and A-4L) infecting filamentous heterocystous cyanobacteria from the order Nostocales and non-heterocystous from order Oscillatoriales, plus those infecting marine *Synechococcus* spp. and *Prochlorococcus* spp. The DNApol tree (**Figure 6**) showed the phage TC-CHP58 as part of a monophyletic clade with all cyanopodoviruses described as infecting freshwater filamentous cyanobacteria, and more distantly, with the marine cyanopodovirus clade that infects *Synechococcus* spp. and *Prochlorococcus* spp. Both cyanophage subgroups are closely related with podoviruses from the Autographivirinae subfamily, which includes all T7 relatives. Furthermore, the phylogeny of the MCP was constructed for freshwater and marine representatives of the Autographivirinae subfamily. The available MCP gene from BHS3 Cyanophage partial genome, that is the only known thermophilic representative within the Podoviridae family, was also included (Zablocki et al., 2017). The MCP tree (**Supplementary Figure S4**) showed similar results to the DNApol tree (**Figure 6**), with a monophyletic origin for all freshwater cyanophages infecting filamentous cyanobacteria, emphasizing the division between freshwater and marine cyanobacterial viruses, and their affiliation with T7 phage. The thermophilic representatives of Podoviridae family were located in different branches inside the freshwater clade, with BHS3 more basal than TC-CHP58.

### CRISPR Arrays on TC-CHP58 Host

Given the high abundance (Mackenzie et al., 2013; Alcamán et al., 2015) and activity (Alcamán et al., 2015) of cyanobacteria, such as *Mastigocladus* spp., in Porcelana hot spring (**Figure 3A**), and in order to confirm the putative host of phage TC-CHP58, CRISPR spacer arrays were identified using the CRISPRFinder tool (Grissa et al., 2007) for seven *Mastigocladus* spp. Contigs, obtained from metagenome assemblies at 48°C, 58°C, and 66°C. Three CRISPR *loci* were common between all temperatures (48_CRISPR_2, 58_CRISPR_5, and 66_CRISPR_2), while four *loci* were specific to higher temperatures (58–66°C) (**Table 2**). In total, the seven CRISPR *loci* contain 562 spacers, of which 25 of them had a proto-spacer sequence in the TC-CHP58 genome (**Table 2**). From the 25 spacers, 19 have a target ORF of known function, such as DNA polymerase, dTMP, portal protein, M23-petidase, tail protein, tail fiber, and deoxycytidine triphosphate deaminase. In general, each CRISPR *loci* contained spacers against different ORFs on TC-CHP58, or even against different locations on the same ORF. For the 25 spacers, searching the nt/nr database, using BLASTN and BLASTX, showed no similarity to any know sequence. Finally, in order to check if CRISPR systems were active, expression of the seven *loci* was directly quantified in the three metatranscriptomes. For all temperatures, slightly lower transcript levels were found compared to the *Mastigocladus* RUBISCO gene (**Figure 5**).

### Identifying Single Nucleotide Variants in TC-CHP58 Genome

To assess if mismatches between the CRISPR spacer and proto-spacer sequences in TC-CHP58 genome were concealing potential variations in TC-CHP58 populations, a SNV calling was conducted. For this task LoFreq tool was used, as it is high sensitivity and has low false positive rates, lower as <0.00005% (Wilm et al., 2012) and higher as 8.3% (Huang et al., 2015). This approach, together with the use of sequences with qualities over q28 (whose error probability in the base call is ≤1.58%), allow us to consider these SNVs as real mutations.

A different number of SNVs was found at each temperature. TC-CHP58 showed 1611, 930, and 671 variant sites at 48°C, 58°C, and 66°C, respectively, unevenly distributed throughout the viral genome (**Supplementary Figure S5**). Considering the three metagenomes, a total of 3212 variable sites were present in the TC-CHP58 genome, with 391 SNVs present over all temperatures (**Supplementary Figure S5**). Most of the SNVs (74% on average) were located at coding regions on the TC-CHP58 genome, with variable rates, ranging from 15 to 0 SNVs for each 100 bp (**Supplementary Table S5**) over different ORFs.

A detailed analysis of SNVs in CRISPRs proto-spacer sites revealed the presence of these polymorphisms in 14 of the 25 spacer targets, with 13 mismatches and 4 perfect matches (**Table 2**). The total number of polymorphic sites was 22, with 13 SNVs causing a synonymous substitution and 7 causing a non-synonymous substitution (**Table 2**).

**Table 2:** CRISPR *loci* at each temperature detailed information and SNVs analysis for TC-CHP58 proto-spacer including alleles frequency and SNV coding effect.

T°C Sample	Virotope Sequence	Viral target	CRISPR *loci*	Proto-spacer Start	Proto-spacer End	Mismatch position	SNV position	Alleles ^∗^	Frequency of in CRISPR allele	SNV effect	Codon change
48	ACCTTTCAGACCTAACTC TAAAGTTACTATCACAGAT	Internal protein-M23-peptidase	48_CRISPR_2_NODE_1554	26558	26594	26564; 26567; 26582	–	–	–	–	–
58	AGAAGTTTTTCTTCGCCAA GATATATGGTGCTGGTCTAA	DNA polymerase	58_CRISPR_10_NODE_13413	282	320	–	282; 292; 313	G/T; C/A; G/A	0.079; 0.552; 0.549	All silent	CGA/AGA; GGG/CGT; TTC/TTT
58	GTGTTGGTGCTCTTGGAGT ACCGTTCAGAATAGGT	Hypothetical protein	58_CRISPR_10_NODE_13413	35908	35942	–	35908	G/A	0.052	Silent	GGC/GGT
58	AGTTGTGCCCCTTGAGCTA GAGAATTTGCTGCACCT	Internal protein-M23-peptidase	58_CRISPR_10_NODE_13413	24692	24727	–	–	–	–	–	–
58	TAAACTGGTCGGGATTGTG TACATTCCATGCACTC	NC	58_CRISPR_10_NODE_13413	8740	8774	–	8753	C/G	0.53	–	–
58	ACTATCTGATCAAACCGGG GCTACACGGTAAATCGTTAGA	Tail fiber protein	58_CRISPR_10_NODE_13413	36649	36688	36650	36675	C/T	0.522	A/V	GCT/GTT
58	ACCTTTCAGACCTAACTCT AAAGTTACTATCACAGAT	Internal protein-M23-peptidase	58_CRISPR_5_NODE_1091	26558	26594	26565; 26567; 26582	–	–	–	–	–
58	CCCAACAACGTCTAAATAAA TCTTTCTATGATATGC	Hypothetical protein	58_CRISPR_8_NODE_4438	24219	24254	24226; 24229; 24238	–	–	–	–	–
58	AATACGGTTGTAGTACTCTT GAAGAGGTGTTACCG	Hypothetical protein	58_CRISPR_8_NODE_4438	30971	31005	30972	30978	G/A	0.588	Silent	ACG/ACA
58	GAAAGGGTAAGGTGTCAAA ATTGGGATTATTAGTGTTAG	Internal protein-M23-peptidase	58_CRISPR_8_NODE_4438	27172	27210	–	27180; 27181; 27186	T/A; C/A; A/C	0.626; 0.613; 0.575	S/T; S/Y; T/P	TCT/ACT; TCT/TAT; ACC/CCC
58	GCATTAATCGCGGGGTTAG GGTGATACCACCTA	Tail protein	58_CRISPR_8_NODE_4438	34211	34243	34214; 34226; 34241	–	–	–	–	–
58	TAGCTTAACATTACCACAG GGGATAAGCTGTTGTATATCC	Deoxycytidine triphosphate deaminase	58_CRISPR_9_NODE_4711	38225	38264	38264	38225; 38261	C/T G/A	0.057; 0.046	All silent	CTG/CTA; GAC; GAT
58	GACTTGATCTTTTCCGCT TTCTTGTAGCGCAGTATCTT	DNA polymerase	58_CRISPR_9_NODE_4711	668	705	–	671; 673	C/T; T/G	0.031; 0.040	R/K; Silent	AGG/AAG
58	ACGGGGTTGATCTTCCCCG CGAAGTGGTTGTCACCGAAT	dTMP kinase	58_CRISPR_9_NODE_4711	6338	6376	6375	6350	G/C	0.575	K/N	AAG/AAC
58	AAATACATCCCCCACTTTAG GAGGTAACCCCAC	Hypothetical protein	58_CRISPR_9_NODE_4711	36127	36159	–	–	–	–	–	–
58	ACAGCGAAAGCAATTTGTC TCTGAGGCTAACAAGTT	Internal protein-M23-peptidase	58_CRISPR_9_NODE_4711	25742	25777	25776	–	–	–	–	–
58	GTCGTATCTCAATGTACTCT TTGTAGTCTTTCCA	Internal protein-M23-peptidase	58_CRISPR_9_NODE_4711	25917	25950	–	25946	C/A	0.041	Silent	ATC/ATA
58	CAATCACACCTAACCCCAT AGGTGACCGCACAACA	Portal protein	58_CRISPR_9_NODE_4711	15920	15954	–	15941; 15953	A/G; A/T	0.651; 0.055	All silent	GGA/GGG; ATA/ATT
58	TAGCTGATTGGAAAGCAGA CGCTGGATTATTACAC	Tail protein	58_CRISPR_9_NODE_4711	33859	33893	–	–	–	–	–	–
66	ATCTGTGATAGTAACTTTAG AGTTAGGTCTGAAAGGT	Internal protein-M23-peptidase	66_CRISPR_2_NODE_1045	26558	26594	26566; 26567; 26582	–	–	–	–	–
66	TTAGACCAGCACCATATATC TTGGCGAAGAAAAACTTCT	DNA polymerase	66_CRISPR_3_NODE_1491	282	320	282	292; 313	C/A; G/A	0.437; 0.024	All silent	GGG/CGT; TTC/TTT
66	ACCTATTCTGAACGGTACTCCA AGAGCACCAACAC	Hypothetical protein	66_CRISPR_3_NODE_1491	35908	35942	35908	–	–	–	–	–
66	AGGTGCAGCAAATTCTCTAGC TCAAGGGGCACAACT	Internal protein-M23-peptidase	66_CRISPR_3_NODE_1491	24692	24727	–	–	–	–	–	–
66	GAGTGCATGGAATGTACA CAATCCCGACCAGTTTA	NC	66_CRISPR_3_NODE_1491	8740	8774	–	8753	C/G	0.051	–	–
66	TCTAACGATTTACCGTGTAGC CCCGGTTTGATCAGATAGT	Tail fiber protein	66_CRISPR_3_NODE_1491	36649	36688	36650	36675	C/T	0.056	A/V	GCT/GTT

*^∗^Virus allele/CRISPR allele*.

## Discussion

The study of viruses from thermophilic phototrophic microbial mat communities remains largely unexplored except for a few cases providing limited information on viral presence within these communities (Heidelberg et al., 2009; Davison et al., 2016). Thus far, no study has characterized viral composition and activity, or the identity of any complete viral genome. Here, using metagenomic and metatranscriptomic approaches, the composition of the most abundant and active viruses associated with the dominant members of the thermophilic bacterial community have been characterized, describing for the first time a full genome from a thermophilic cyanopodovirus (TC-CHP58). Moreover, the active cross-fire between this new cyanophage and its host is demonstrated, through TC-CHP58 population diversification (SNV), and *Mastigocladus* spp. CRISPR heterogeneity, as a response to selective pressure from the host defense system and viral predation, respectively.

### Active and Ubiquitous Cyanophage-Type Caudovirales in Phototrophic Microbial Mats

The taxonomic classification of small subunit rRNA (**Supplementary Table S1**) indicates that the phototrophic mats in Porcelana hot spring are dominated by Bacteria (96% on average) as commonly observed in other thermophilic phototrophic microbial mats (Inskeep et al., 2013; Bolhuis et al., 2014).

Porcelana microbial mats are mainly built by filamentous representatives of two phototrophic phyla, Cyanobacteria (oxygenic) and Chloroflexi (anoxygenic), with *Mastigocladus, Chloroflexus*, and *Roseiflexus* as the main genera, respectively. This is verified by previous surveys carried out by the authors (Mackenzie et al., 2013; Alcamán et al., 2015), as well as investigations from the White Creek, Mushroom, and Octopus hot springs in Yellowstone (Miller et al., 2009; Inskeep et al., 2013; Klatt et al., 2013; Bolhuis et al., 2014), presenting similar pH, thermal gradient and low sulfide concentrations.

Porcelana dominant viruses (∼70% and ∼68% of metagenomic and metatranscriptomic reads) are from the families Myoviridae, Podoviridae, and Siphoviridae within the Caudovirales Order (**Figure 2**), which typically infect Bacteria and some non-hyperthermophilic Archaea (Maniloff and Ackermann, 1998). These results were also supported by TEM images (**Figure 1**). The small decrease in transcripts associated to caudovirales with the increase in temperature is due to the reduction of sequences related to Podovirus and Myovirus families. A plausible explanation, is that at high temperatures some representatives of these families might have a lysogenic lifestyle, then a fraction of them will remain inactive as prophages.

Dominance by Caudovirales was only reported recently from the Brandvlei hot spring, South Africa, a slightly acidic (pH 5.7) hot spring with moderate temperature (60°C) and green microbial mat patches (Zablocki et al., 2017). Previously, the presence of this viral order had only been suggested in moderate thermophilic phototrophic mats from Yellowstone hot springs, through indirect genomic approximations, such as spacers in CRISPR *loci*, from dominant bacterial members (Heidelberg et al., 2009; Davison et al., 2016) or classifications based on nucleotide motives in metaviromic data (Pride and Schoenfeld, 2008; Davison et al., 2016).

Contributions from megavirus sequences were also identified in Porcelana hot spring (**Figure 2**), with an average of ∼24% viral metagenomic reads, associated with unicellular eukaryotic hosts such as those from Phycodnaviridae and Mimiviridae families, and also the family Marseilleviridae, but to a lesser extent. The presence of VLPs from these three viral families could not be corroborated through TEM, using the limited available viral fraction (<0.2 μm) within the community, as it has been previously documented that nucleocytoplasmic large DNA viruses (NCLDV) particles are only found in larger viral fractions (Pesant et al., 2015). The ubiquity of NCLDVs in hot springs was previously described in a hydrothermal freshwater lake in Yellowstone, with assemblies of genomes from Phycodnaviridae and Mimiviridae (Zhang et al., 2015).

Viral relative abundances and activity reported here can be affected by the lack of replicates at this highly local heterogeneity samples. However, the fact of having three different temperature sampling points for metagenomics and metatranscriptomics, partially compensates the replicate limitation.

Furthermore, many viruses in an environmental sample share a degree of similarity in their genomic sequence, and this intrinsic complexity of metagenomic/metatranscriptomic samples makes difficult to accurately estimate the relative abundances or activity of specific phages at low ranks of taxonomy tree, such as the species level (Sohn et al., 2014). To avoid this problem, our strategy focused on the use of the LCA algorithm at higher taxonomic levels (Order and Family) to classify the viral reads, as well as for the inferred hosts, we use the phylum level.

Virus-host inference in Porcelana phototrophic mats (**Figure 3B**), demonstrated that the most frequent targets for viral infections were the most dominant and active components of the bacterial communities. Similarly, this is the case in other environments, such as in the human microbiome (Macklaim et al., 2013) and marine communities (Thingstad et al., 2014; Zeigler-Allen et al., 2017). In Porcelana, it is demonstrated that within microbial mats at 48°C and 58°C, cyanophages were among the most active viruses (**Figure 3B**), as were Cyanobacteria, such as *Mastigocladus* spp., as exemplified in terms of primary production and nitrogen fixation (Alcamán et al., 2015). The presence of cyanophages has been previously suggested in Yellowstone hot spring phototrophic mats (Heidelberg et al., 2009; Davison et al., 2016), and more recently in the Brandvlei hot spring, South Africa (Zablocki et al., 2017). Heidelberg et al. (2009) found that CRISPR spacers in unicellular cyanobacteria *Synechococcus* isolates (Syn OS-A and Syn OS-B9) from Octopus Hot Spring, might have 23 known viral targets (lysozyme-related reads, PFAM DUF847) on an independently published metavirome from the same hot spring. More recently, 171 viral contigs associated with the host genus S*ynechococcus*, based on tetranucleotide frequencies, were identified from a microbial mat (60°C) metavirome from Octopus Spring. The majority of the annotated ORFs on the viral contigs coded for glycoside hydrolases, with lysozyme activity, identifying six CRISPR proto-spacers in those genes (Davison et al., 2016). Even though a taxonomic relationship with cyanophages was not confirmed for those proto-spacers containing contigs (Heidelberg et al., 2009; Davison et al., 2016), it provides evidence toward the presence of cyanophages related sequences within these thermophilic mats. The work by Zablocki et al. (2017) reconstructed a 10 kb partial genome of a new cyanophage (BHS3) from Brandvlei hot spring metavirome, stating that cyanophages appear to be the dominant viruses in the hot spring. The BHS3 contig (MF098555) contains nine ORFs, with the majority of the identified proteins having a close relation to the Cyanophage PP and *Phormidium* phage Pf-WMP3, which infect freshwater filamentous cyanobacteria *Phormidium* and *Plectonema*.

The presence of cyanophages related sequences in thermophilic phototrophic mats is significant, since these viruses are known to play an important role in the evolution of cyanobacteria (Shestakova and Karbysheva, 2015). Cyanophages affect the rate and direction of cyanobacterial evolutionary processes, through the regulation of abundance, population dynamics, and natural community structure. This has been extensively studied and demonstrated for marine environments (Weinbauer and Rassoulzadegan, 2004; Avrani et al., 2011). These cyanophages are proven to play a relevant role in the marine biogeochemical cycles, through the infection and lysis of Cyanobacteria, affecting carbon and nitrogen fixation (Suttle, 2000). Moreover, cyanophages act as a global reservoir of genetic information, as they are vectors for gene transfer, meaning that cyanobacteria can acquire novel attributes within aquatic environments (Kristensen et al., 2010; Chénard et al., 2016).

Caudoviruses were prevalent at 66°C in Porcelana, and potentially infecting Firmicutes, Proteobacteria, and Actinobacteria. These phila have also been previously identified in other hot springs at temperatures above 76°C, such as in Octopus and Bear Paw (Pride and Schoenfeld, 2008). At high temperatures in Porcelana also the phylum Chloroflexi was dominant in the phototrophic mat (**Figure 3A**). However, viral sequences related to this taxon could not be retrieved, as neither viruses nor viral sequences have been confirmed to infect members of this phylum in any environment. Davison et al. (2016), described viral contigs associated with *Roseiflexus* sp. from a metavirome from Octopus Spring, but only raw reads are publicly available, without taxonomic assignation. Finally, the recently released IMG/VR database (Paez-Espino et al., 2016) contains three contigs associated by CRISPR spacers to *Chloroflexus* sp. Here, a BLASTP analyses against RefSeq viral proteins revealed that six of these proteins have a best hit in *Mycobacterium* phage proteins and one which best hit was a *Clavibacter* phage protein. These findings, suggest that some of the viral reads classified as Actinobacteria viruses could be instead from unknown Chloroflexi viruses.

### Viral Mining Reveals a New Infective Thermophilic Cyanopodovirus Lineage

Metagenomic surveys of viral genomes are an effective way to detect unknown viruses (Roux et al., 2015a,b; Zhang et al., 2015; Voorhies et al., 2016). In metagenomics, two key elements for virus detection are the presence of viral hallmark genes and the circularity of viral contigs (Roux et al., 2015a,b). Based on these two principles, a complete genome (TC-CHP58) was identified. The genome was represented by a viral contig of 50 kb, which is a typical size for Caudovirales members from the Podoviridae family. The genome size and viral core proteins affiliated with the Podovirus seems to make TC-CHP58 the first report of a full genome of a thermophilic cyanopodovirus. Moreover, the genome organization (**Figure 4B**) shows a consistent synteny with other cyanopodoviruses, which also lack RNA polymerase inside the T7 supergroup, as described for the viruses Pf-WMP4, Pf-WMP3, Cyanophage PP, Anabaena phage A-4L (Liu et al., 2007, 2008; Zhou et al., 2013; Ou et al., 2015), and the recently reported partial genome of the thermophilic BHS3 cyanophage (Zablocki et al., 2017). Initially, the presence of a single-subunit RNA polymerase that binds phage specific promoters was considered to be a major, and unique characteristic of the T7 supergroup (Dunn et al., 1983). However, more recently, it has been proposed that podoviruses that share extensive homology with T7, but lack the phage RNA polymerase, are still part of the T7 supergroup, as distant and probably ancient branches (Hardies et al., 2003).

TC-CHP58 presented a genome organization that can be divided into two portions (**Figure 4A**); with ORFs in the sense strand related to DNA replication and modification, and genes encoded in the antisense strand related to virion assembly. This genome organization is also present in other freshwater T7-related podoviruses that infect filamentous cyanobacteria (Liu et al., 2007, 2008; Zhou et al., 2013; Ou et al., 2015), including the thermophilic BHS3 cyanophage (Zablocki et al., 2017). This setup is also similar to the class II and III organization genes in T7-like viruses, where class II genes are responsible for DNA replication and metabolism, and class III genes include structural and maturation genes (Dunn et al., 1983). The VIRFAM analysis of neck protein organization verifies the classification of TC-CHP58 within the Podoviridae family (**Supplementary Figure S3**), where the Type 3 podovirus encompasses T7-like phages from Autographivirinae subfamilies and several other genera (Lopes et al., 2014). The T7-like classification for TC-CHP58, and other podoviruses that infect freshwater filamentous cyanobacteria, is supported by the organization of the genome into two portions as well as the organization of the neck proteins.

The phylogenetic position of TC-CHP58, based on DNA polymerase I (DNApol) (**Figure 6**) and MCP (**Supplementary Figure S4**) predicted proteins, confirm the affiliation of this new virus within the family Podoviridae. Both phylogenetic markers verify the separation between the marine from the freshwater cyanopodoviruses within the T7 family, as previously proposed (Liu et al., 2007; Ou et al., 2015). These results also support the connection between the T7 phages and marine and freshwater cyanopodoviruses (Chen and Lu, 2002; Hardies et al., 2003; Liu et al., 2007; Ou et al., 2015), including TC-CHP58 and BHS3 as representatives of a novel, and potentially globally distributed thermophilic cyanophage lineage. Moreover, this data demonstrates that marine and freshwater cyanopodoviruses, including the thermophilic TC-CHP58, are part of the Autographivirinae subfamily as previously suggested for Cyanophage P60 and Roseophage SIO1 (Labonté et al., 2009), both included in this analysis.

In Porcelana, the virus host ratio relating to TC-CHP58 presence was lower than the typical values observed in freshwater environments (Maranger and Bird, 1995), being more similar to other geothermal environments where viral density is typically lower, with 10- to 100-fold less viruses than host cells (López-López et al., 2013). This is expected, considering that there are abundant cyanobacteria in phototrophic mats in Porcelana in comparison with the 10^4^ mL^-1^ VLPs observed in the water of hot springs (Breitbart et al., 2004). It is also demonstrated that TC-CHP58 presented higher infection efficiency, as revealed by the viral DNA to RNA ratios at lower temperatures (58°C, then 48°C) with cyanobacteria dominating, while at 66°C most of the TC-CHP58 remained inactive (**Figure 5**). Infection inefficiency is multidimensional, as it initiates from reduced phage adsorption, RNA, DNA, and protein production (Howard-Varona et al., 2017). Thus, the high copy number of TC-CHP58 DNA at 66°C may be due to the persistence of viral DNA (Mengoni et al., 2005) encapsidated extracellularly and intermixed in the microbial mat were the host (*Mastigocladus* spp.) has a low activity as evidenced by the low expression of the RUBISCO gene and the CRISPR *loci*. An alternative explanation is the absence, or the diminished presence, of the specific host due to intraspecific diversification as evidenced by the existence of different CRISPR *loci* at different temperatures. This theory has been proposed for other cyanobacteria, such as *Prochlorococcus* and *Phormidium*, where slight differences in fitness, niche, and selective phage predation, explain the coexistence of different populations (Kashtan et al., 2014; Voorhies et al., 2016). The last explanation acquires special importance in light of recent evidence that variations in the structure and function of the heterocyst and differential CRISPR *loci* are fundamental to diversification of *Mastigocladus laminosus* (also known as *Fischerella thermalis*), a cosmopolitan thermophilic cyanobacterium, reinforcing the importance of viral predation (Sano et al., 2018).

### CRISPR Spacers Assign *Mastigocladus* spp. as Putative Hosts for TC-CHP58

It was possible to verify *Mastigocladus* spp. as putative hosts for the new cyanopodovirus (TC-CHP58), via the analysis of CRISPR spacers found in the cyanobacteria, recovered from contigs obtained in the same metagenomic datasets. This methodology has been previously used for the identification of novel viruses in hot springs (Heidelberg et al., 2009; Snyder et al., 2010; Davison et al., 2016), as well as in other environments such as acid mines (Andersson and Banfield, 2008), the human microbiome (Stern et al., 2012), as well as sea ice and soils (Sanguino et al., 2015).

Observations from the CRISPR *loci* over all temperatures (**Table 2**) indicated that, in general, proto-spacers in the TC-CHP58 genome were distributed on coding, and therefore more conserved regions. The expression of seven CRISPR *loci* (**Figure 5**), demonstrated the activity of the *Mastigocladus* spp. defense system against TC-CHP58 over all temperatures. CRISPR arrays are transcribed into a long precursor, containing spacers and repeats, that are processed into small CRISPR RNAs (crRNAs) by dedicated CRISPR-associated (Cas) endoribonucleases (Brouns et al., 2008). Although it is not possible to measure mature crRNAs, as due to their small size they are likely to be filtered out in RNA-seq libraries, this approximation has been validated using large datasets (Ye and Zhang, 2016).

Despite variations in the number of CRISPR *loci* observed at each temperature, with 60% of the total CRISPR *loci* found in Mastigocladus contigs at 58°C, the abundance of reads agreed with the abundance of other genes required by these cyanobacteria, such as the RUBISCO gene (**Figure 5**). This further verified that the *loci* are from *Mastigocladus* populations. The different CRISPR *loci* found over the different temperatures in Porcelana (**Table 2**), also reinforces the notion that diversification of *Mastigocladus* is partly due to selective pressure exerted by the predation of viruses, such as TC-CHP58. This theory has been previously put forward for *Mastigocladus laminosus* in Yellowstone (Sano et al., 2018), and proposed for marine cyanobacteria (Rodriguez-Valera et al., 2009; Kashtan et al., 2014).

Furthermore, each CRISPR *loci* contains spacers that corresponds to different proto-spacers in the TC-CHP58 genome. Increases in spacer number and diversity against the same virus may explain the increase in interference, whilst decreasing the selection of escape mutants (Staals et al., 2016). Priming mechanisms are the most efficient form of obtaining new spacers (Staals et al., 2016), using a partial match between a pre-existing spacer and the genome of an invading phage to rapidly acquire a new “primed” spacer (Westra et al., 2016). Then, over-representation of spacer sequences in some regions of the TC-CHP58 genome may be related to a site that has already been sampled by the CRISPR-Cas machinery or by other biases such as the secondary structure of phage ssDNA, GC content, and transcriptional patterns (Paez-Espino et al., 2013).

The selection pressure of multiple spacers in *Mastigocladus* CRISPR *loci* leads to the emergence of SNVs in the TC-CHP58 viral populations (**Table 2**), which cause mismatches between spacers and proto-spacers, resulting in the attenuation or evasion of the host immune response (Shmakov et al., 2017). It is still possible to utilize mismatched spacers for interference and/or primed adaptation, however, the degree of tolerance to mismatches for interference among the CRISPR-Cas, varies substantially between different CRISPR-Cas type systems (Shmakov et al., 2017). The variable frequency (0.6–0.02) of the corresponding spacer SNVs alleles on TC-CHP58 proto-spacers, suggests that some variants are more prevalent throughout the population, regardless of whether the SNV causes a silent mutation. Based on this evidence, it has been proposed that, for other microbial communities, only the most recently acquired spacer can exactly match the virus. This suggests that community stability is driven by compensatory shifts in host resistance levels and virus population structure (Andersson and Banfield, 2008).

The present study describes the underlying viral community structure and activity of thermophilic phototrophic mats. Moreover, abundant virus populations are linked to dominant bacteria, demonstrating the effectiveness of omics approaches in estimating the importance and activity of a viral community, in this case with thermophilic cyanophages.

Additionally, the first full genome of a new T7-related virus that infects thermophilic representatives of the cyanobacterium *Mastigocladus* spp. was here retrieved. This genome may represent a novel, globally present, freshwater thermophilic virus from a new lineage from the Podoviridae family. The latter was strongly suggested by the significant phylogenetic relationship and shared gene organization with the BHS3 cyanophage partial genome (South Africa). Even more, TC-CHP58 proteins also matches several contigs that include common viral hallmarks genes in the IMG/VR database. However, further work is necessary to fully understand the global representation and relevance of this virus, which complete genome is presented here as first reference available.

Finally, the evolutionary arms race between a specific cyanobacteria-cyanophage in the natural environment is exposed, where a there exist a variety of potential scenarios. For instance, host resistance may increase over time forcing the decrease of viral populations, or a specific virus population may occasionally become extremely virulent and cause the crash of the host population as proposed by the “kill the winner” model (Andersson and Banfield, 2008). Alternatively, if CRISPR systems and the diversification of the viral population remain in balance through time, a relatively stable virus and host community may result.

## Data Availability Statement

The datasets generated for this study can be found NCBI as follow: Access to raw data for metagenomes and metatranscriptomes is available through NCBI BioProject ID PRJNA382437. https://www.ncbi.nlm.nih.gov/bioproject/?term=PRJNA382437. The genome of TC-CHP58 has the GenBank accession number KY888885. Contigs containing CRISPRs *loci* have been submitted to NCBI with GenBank accession numbers MG734911 to MG734917.

## Author Contributions

SG-L and BD conceived and designed the experiments. SG-L, OS, and FP performed the experiments. SG-L, CP-A, OS, FP, and BD analyzed the data. SG-L, CP-A, and BD wrote the paper.

## Conflict of Interest Statement

The authors declare that the research was conducted in the absence of any commercial or financial relationships that could be construed as a potential conflict of interest.

## Footnotes

1 sourceforge.net/projects/bbmap/

2 http://beast.bio.ed.ac.uk/Tracer
