# Supplementary material for: Active Crossfire Between Cyanobacteria and Cyanophages in Phototrophic Mat Communities Within Hot Springs
Source: Front Microbiol. 2018 Sep 3;9:2039. doi: 10.3389/fmicb.2018.02039 (PMC6129581; doi:10.3389/fmicb.2018.02039)
Supplement: Supplementary file 1 [file Data_Sheet_1.PDF]

Supplementary Material

Active crossfire between Cyanobacteria and Cyanophages in phototrophic mat communities within hot springs

Sergio Guajardo-Leiva, Carlos Pedrós-Alió, Oscar Salgado, Fabián Pinto, and Beatriz Díez

*** Correspondence:** Beatriz Díez: bdiez@bio.puc.cl

1 Supplementary Figures and Tables

1.1 Supplementary Figures

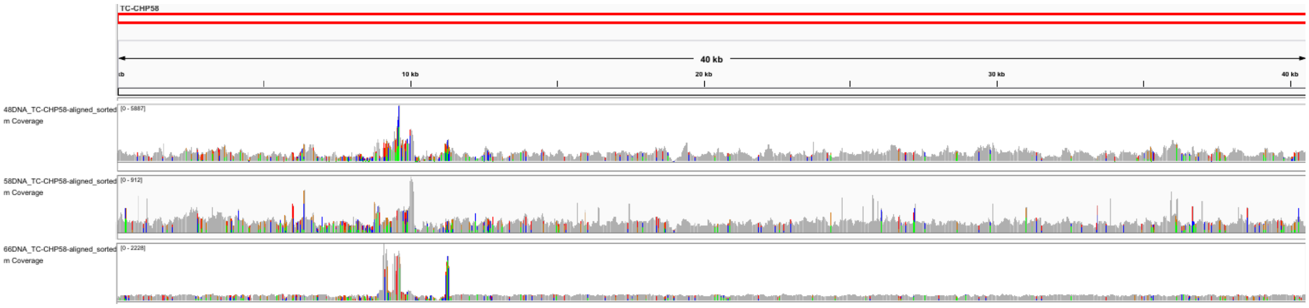

Supplementary Figure S1. Reads recruitment to TC-CHP58 genome from metagenomes of Porcelana hot spring temperature gradient. Grey lines represent zones of perfect match and colored lines represent different ratios of nucleotide mismatched positions.

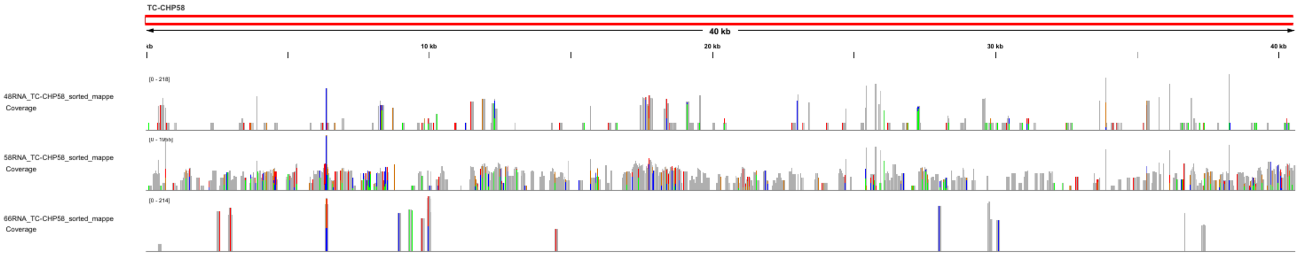

Supplementary Figure S2. Reads recruitment to TC-CHP58 genome from metatrancriptomes of Porcelana hot spring temperature gradient. Grey lines represent zones of perfect match and colored lines represent different ratios of nucleotide mismatched positions. White zones represent absence of mapping.

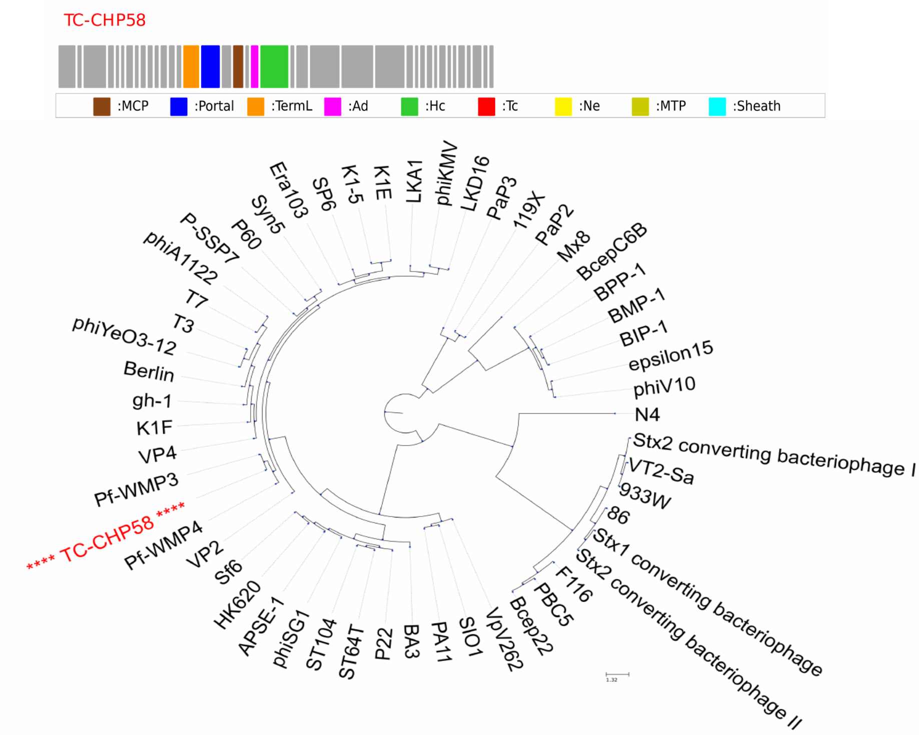

Supplementary Figure S3. TC-CHP58 VIRFAM neck protein organization analyses. Upper panel show a schematic representation of TC-CHP58 gene organization. Bottom panel show a hierarchical clustering of neck proteins of TC-CHP58 and other related viruses.

Supplementary Material

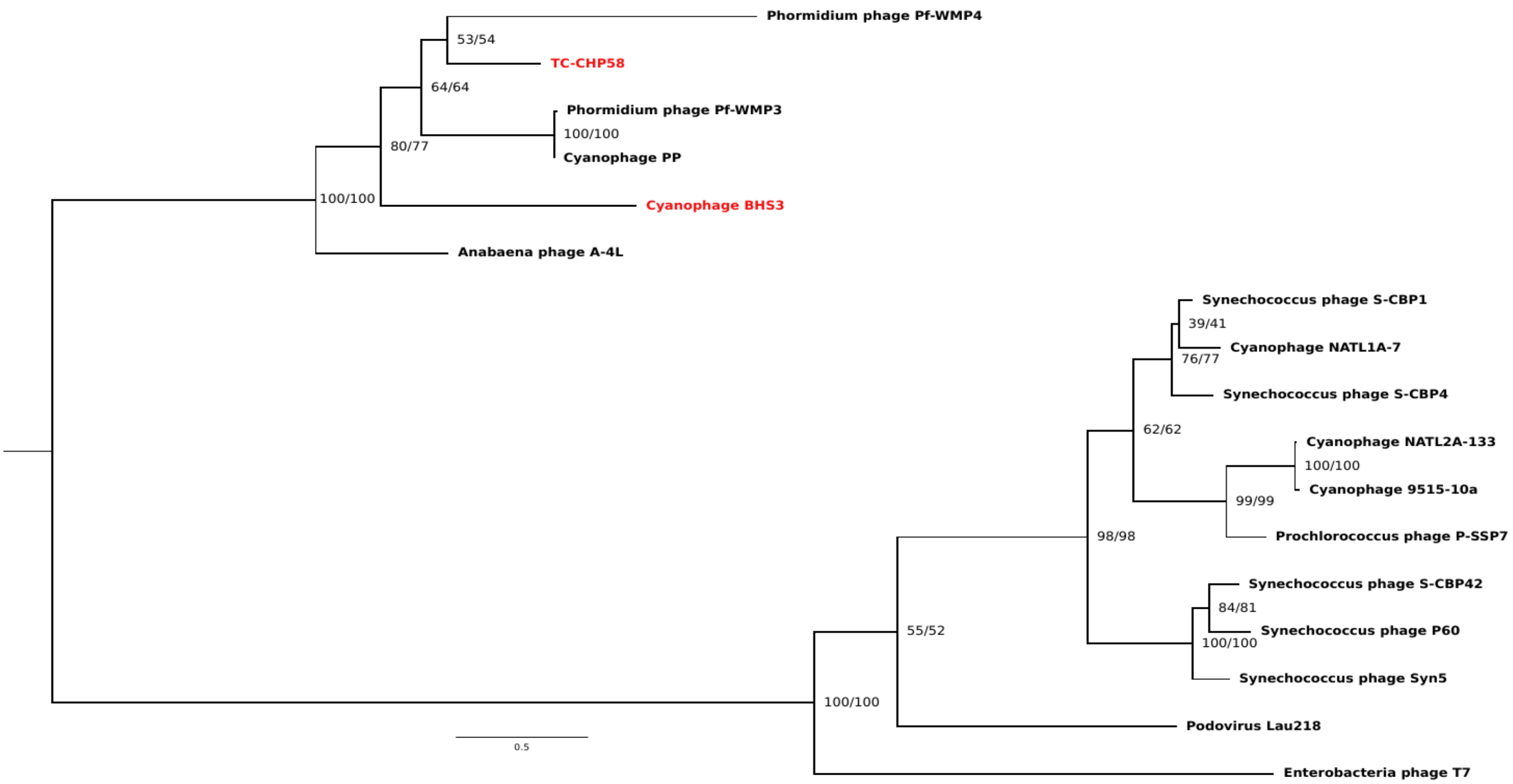

Supplementary Figure S4. Maximum likelihood phylogenetic gene tree of Major Capsid protein. Numbers indicate Ultra fast bootstrap (UFBoot) values. Only UFBoot values over 50 are show. The sequence characterized in the present work is reported in red bold letters. Scale bar: 0.6 amino acid substitutions per site.

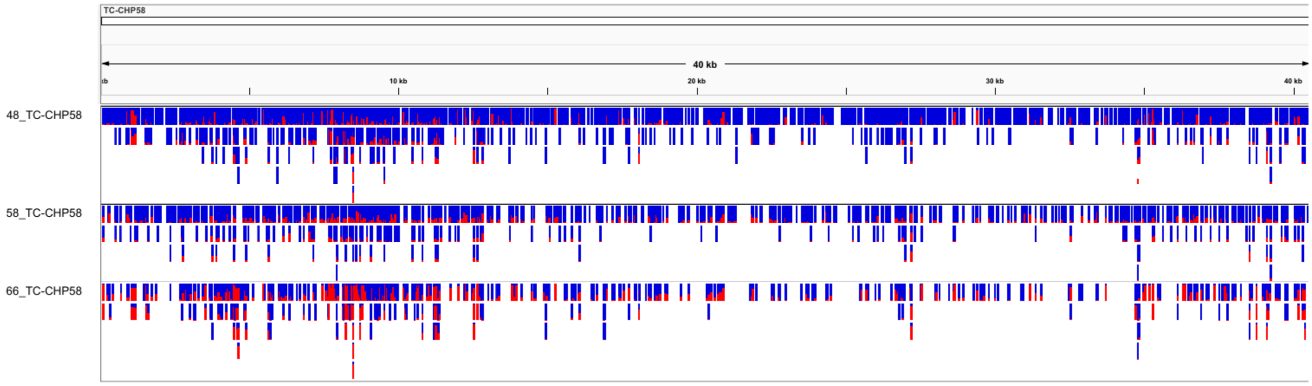

Supplementary Figure S5. SNVs calling along TC-CHP58 genome for TC-CHP58 populations over Porcelana temperature gradient. Red and blue lines represent the ratio of different alleles for each SNV.

1.2 Supplementary Tables

Supplementary Table S1. Summary information about sequencing depth, quality filtering, read mapping and assembly of Porcelana metagenomes an metatranscriptomes.

Sample	Raw reads (10E6)	Raw bases (10E6)	Reads (10E6) after quality filter	Bases (10E6) after quality filter	Assembled reads (10E6)	Assembled bases (10E6)	VNA aligned reads (10E6)	VNA aligned bases (10E6)	Bacteria 16S aligned reads	Archaea 16S aligned reads	Eukarya 18s aligned reads
48DNA	336.8	42448.92	279.37	21886.89	46.43	5585.51	1.1	123.23	147241	1981	683
58DNA	140.12	17655.26	118.83	10263.5	34.83	4176.35	0.559	62.36	90911	95	473
66DNA	10.5	1130.51	7.143	684.32	6.28	604.54	0.044	5.39	6928	4	396
48RNA	15.5	1953.23	8.4	1035.14	--	--	0.312	38.9	--	--	--
58RNA	38.5	4850.86	22.71	2819.89	--	--	0.472	59.05	--	--	--
66RNA	149.2	18805.33	52.12	6866.81	--	--	0.185	23.3	--	--	--

Supplementary Material

Supplementary Table S2. Accession numbers of sequences used in phylogenetic analyses of DNA Polymerase, and Major Capsid of TC-CHP58

DNApol Accession number	Organism	Capsid Accession number	Organism
YP_001285436.1	Synechococcus phage Syn5	NP_041998.1	Enterobacteria phage T7
AAG02598.1	Roseobacter phage SIO1	YP_001285448.1	Synechococcus phage Syn5
AAL73268.1	Synechococcus phage P60	YP_001285797.1	Phormidium phage Pf-WMP3
AAO73157.1	Pseudomonad phage gh-1	YP_005087431.1	Cyanophage 9515-10a
ABG75891.1	Phormidium phage P4	YP_005087453.1	Cyanophage NATL1A-7
ABU50233.1	Cyanophage S-CBP1	YP_005087541.1	Cyanophage NATL2A-133
ABU50234.1	Cyanophage S-CBP3	YP_008766991.1	Cyanophage PP
ABU50235.1	Synechococcus phage S-CBP42	YP_009042168.1	Podovirus Lau218
CAA24412.1	Enterobacteria phage T7	YP_009042803.1	Anabaena phage A-4L
CAB63614.1	Yersinia phage phiYeO3-12	YP_009103190.1	Synechococcus phage S-CBP1
CAC86283.1	Enterobacteria phage T3	YP_009103797.1	Synechococcus phage S-CBP4
NP_044817.1	Streptococcus phage Cp-1	YP_009173806.1	Synechococcus phage P60
NP_049662.1	Enterobacteria phage T4	YP_009220191.1	Synechococcus phage S-CBP42
NP_848283.1	Yersinia phage phiA1122	YP_214206.1	Prochlorococcus phage P-SSP7
YP_001285777.1	Phormidium phage Pf-WMP3	YP_762667.1	Phormidium phage Pf-WMP4
YP_001949769.1	Salmonella phage phiSG-JL2	ASV43892.1	Cyanophage BHS3
YP_002004529.1	Bacillus virus phi29		
YP_002048647.1	Morganella phage MmP1		
YP_002308401.1	Kluyvera phage Kvp1		
YP_006355438.1	Prochlorococcus phage P-SSP7		
YP_006488652.1	Clostridium phage phiZP2		
YP_007006982.1	Clostridium phage phi24R		
YP_008766972.1	Cyanophage PP		
YP_009042789.1	Anabaena phage A-4L		
YP_249581.1	Vibriophage VP4		
YP_338108.1	Enterobacteria phage K1F		
YP_762649.1	Phormidium phage Pf-WMP4		
YP_919001.1	Yersinia phage Berlin		

Supplementary Material

Supplementary Table S3. Relative abundances (%) of Caudovirales community at the host Phylum level, obtained from shotgun sequences in metagenomes (DNA) and metatranscriptomes (RNA), standardized by the total number of reads from each temperature sample.

Temperature ºC	Family	Host-Virus	Percent	Temperature ºC	Family	Host-Virus	Percent	Temperature ºC	Family	Host-Virus	Percent
DNA-48 ºC	Siphoviridae	Actinobacteria	40.2	DNA-48 ºC	Podoviridae	Cyanophyceae	30.5	DNA-48 ºC	Myoviridae	Gammaproteobacteria	36.1
DNA-48 ºC	Siphoviridae	Bacilli	18.7	DNA-48 ºC	Podoviridae	Flavobacteriia	21.2	DNA-48 ºC	Myoviridae	Cyanophyceae	30
DNA-48 ºC	Siphoviridae	Gammaproteobacteria	36.2	DNA-48 ºC	Podoviridae	Gammaproteobacteria	34.7	DNA-48 ºC	Myoviridae	Bacilli	22
DNA-48 ºC	Siphoviridae	Flavobacteriia	2	DNA-48 ºC	Podoviridae	Bacilli	4.1	DNA-48 ºC	Myoviridae	Actinobacteria	3.5
DNA-48 ºC	Siphoviridae	Alphaproteobacteria	0.6	DNA-48 ºC	Podoviridae	Actinobacteria	3.5	DNA-48 ºC	Myoviridae	Clostridia	2.5
DNA-48 ºC	Siphoviridae	Verrucomicrobia	0.8	DNA-48 ºC	Podoviridae	BetaProteobacteria	3.5	DNA-48 ºC	Myoviridae	Betaproteobacteria	3.1
DNA-48 ºC	Siphoviridae	Methanobacteria	0.2	DNA-48 ºC	Podoviridae	Enviromental	2.4	DNA-48 ºC	Myoviridae	Alphaproteobacteria	2.7
DNA-48 ºC	Siphoviridae	Clostridia	0.1	DNA-58 ºC	Podoviridae	Cyanophyceae	50.5	DNA-58 ºC	Myoviridae	Gammaproteobacteria	24.8
DNA-48 ºC	Siphoviridae	BetaProteobacteria	1.2	DNA-58 ºC	Podoviridae	Flavobacteriia	22.8	DNA-58 ºC	Myoviridae	Cyanophyceae	44.8
DNA-58 ºC	Siphoviridae	Actinobacteria	52.2	DNA-58 ºC	Podoviridae	Gammaproteobacteria	18.8	DNA-58 ºC	Myoviridae	Bacilli	19.7
DNA-58 ºC	Siphoviridae	Bacilli	25.6	DNA-58 ºC	Podoviridae	Bacilli	3.6	DNA-58 ºC	Myoviridae	Actinobacteria	3.1
DNA-58 ºC	Siphoviridae	Gammaproteobacteria	17.5	DNA-58 ºC	Podoviridae	Actinobacteria	2.1	DNA-58 ºC	Myoviridae	Clostridia	2.6
DNA-58 ºC	Siphoviridae	Flavobacteriia	3	DNA-58 ºC	Podoviridae	BetaProteobacteria	1.3	DNA-58 ºC	Myoviridae	Betaproteobacteria	2.7
DNA-58 ºC	Siphoviridae	Alphaproteobacteria	0.8	DNA-58 ºC	Podoviridae	Enviromental	0.9	DNA-58 ºC	Myoviridae	Alphaproteobacteria	2.4
DNA-58 ºC	Siphoviridae	Verrucomicrobia	0.4	DNA-66 ºC	Podoviridae	Cyanophyceae	23.2	DNA-66 ºC	Myoviridae	Gammaproteobacteria	33.8
DNA-58 ºC	Siphoviridae	Methanobacteria	0.2	DNA-66 ºC	Podoviridae	Flavobacteriia	25.5	DNA-66 ºC	Myoviridae	Cyanophyceae	27.9
DNA-58 ºC	Siphoviridae	Clostridia	0.2	DNA-66 ºC	Podoviridae	Gammaproteobacteria	38.9	DNA-66 ºC	Myoviridae	Bacilli	24.6
DNA-58 ºC	Siphoviridae	BetaProteobacteria	0.1	DNA-66 ºC	Podoviridae	Bacilli	5.7	DNA-66 ºC	Myoviridae	Actinobacteria	4
DNA-66 ºC	Siphoviridae	Actinobacteria	52.6	DNA-66 ºC	Podoviridae	Actinobacteria	2.4	DNA-66 ºC	Myoviridae	Clostridia	4
DNA-66 ºC	Siphoviridae	Bacilli	25.7	DNA-66 ºC	Podoviridae	BetaProteobacteria	3.1	DNA-66 ºC	Myoviridae	Betaproteobacteria	3.1
DNA-66 ºC	Siphoviridae	Gammaproteobacteria	16.8	DNA-66 ºC	Podoviridae	Enviromental	1.3	DNA-66 ºC	Myoviridae	Alphaproteobacteria	2.6
DNA-66 ºC	Siphoviridae	Flavobacteriia	3.2	RNA-48 ºC	Podoviridae	Cyanophyceae	95	RNA-48 ºC	Myoviridae	Gammaproteobacteria	1.5
DNA-66 ºC	Siphoviridae	Alphaproteobacteria	0.8	RNA-48 ºC	Podoviridae	Flavobacteriia	4.3	RNA-48 ºC	Myoviridae	Cyanophyceae	96
DNA-66 ºC	Siphoviridae	Verrucomicrobia	0.3	RNA-48 ºC	Podoviridae	Gammaproteobacteria	0.3	RNA-48 ºC	Myoviridae	Bacilli	1.7
DNA-66 ºC	Siphoviridae	Methanobacteria	0.2	RNA-48 ºC	Podoviridae	Bacilli	0	RNA-48 ºC	Myoviridae	Actinobacteria	0.2
DNA-66 ºC	Siphoviridae	Clostridia	0.2	RNA-48 ºC	Podoviridae	Actinobacteria	0.1	RNA-48 ºC	Myoviridae	Clostridia	0.4
DNA-66 ºC	Siphoviridae	BetaProteobacteria	0.2	RNA-48 ºC	Podoviridae	BetaProteobacteria	0.2	RNA-48 ºC	Myoviridae	Betaproteobacteria	0.1
RNA-48 ºC	Siphoviridae	Actinobacteria	66.5	RNA-48 ºC	Podoviridae	Enviromental	0	RNA-48 ºC	Myoviridae	Alphaproteobacteria	0.2
RNA-48 ºC	Siphoviridae	Bacilli	24.6	RNA-58 ºC	Podoviridae	Cyanophyceae	84	RNA-58 ºC	Myoviridae	Gammaproteobacteria	5
RNA-48 ºC	Siphoviridae	Gammaproteobacteria	7.7	RNA-58 ºC	Podoviridae	Flavobacteriia	14.4	RNA-58 ºC	Myoviridae	Cyanophyceae	88.9
RNA-48 ºC	Siphoviridae	Flavobacteriia	0.7	RNA-58 ºC	Podoviridae	Gammaproteobacteria	0.7	RNA-58 ºC	Myoviridae	Bacilli	4
RNA-48 ºC	Siphoviridae	Alphaproteobacteria	0.5	RNA-58 ºC	Podoviridae	Bacilli	0.3	RNA-58 ºC	Myoviridae	Actinobacteria	0.5
RNA-48 ºC	Siphoviridae	Verrucomicrobia	0	RNA-58 ºC	Podoviridae	Actinobacteria	0.2	RNA-58 ºC	Myoviridae	Clostridia	1
RNA-48 ºC	Siphoviridae	Methanobacteria	0	RNA-58 ºC	Podoviridae	BetaProteobacteria	0.3	RNA-58 ºC	Myoviridae	Betaproteobacteria	0.2
RNA-48 ºC	Siphoviridae	Clostridia	0	RNA-58 ºC	Podoviridae	Enviromental	0.1	RNA-58 ºC	Myoviridae	Alphaproteobacteria	0.3
RNA-48 ºC	Siphoviridae	BetaProteobacteria	0	RNA-66 ºC	Podoviridae	Cyanophyceae	14.6	RNA-66 ºC	Myoviridae	Gammaproteobacteria	33.9
RNA-58 ºC	Siphoviridae	Actinobacteria	69.7	RNA-66 ºC	Podoviridae	Flavobacteriia	43.9	RNA-66 ºC	Myoviridae	Cyanophyceae	19.6
RNA-58 ºC	Siphoviridae	Bacilli	21.1	RNA-66 ºC	Podoviridae	Gammaproteobacteria	20.4	RNA-66 ºC	Myoviridae	Bacilli	27
RNA-58 ºC	Siphoviridae	Gammaproteobacteria	8	RNA-66 ºC	Podoviridae	Bacilli	12.2	RNA-66 ºC	Myoviridae	Actinobacteria	5.5
RNA-58 ºC	Siphoviridae	Flavobacteriia	0.6	RNA-66 ºC	Podoviridae	Actinobacteria	4.2	RNA-66 ºC	Myoviridae	Clostridia	7.9
RNA-58 ºC	Siphoviridae	Alphaproteobacteria	0.3	RNA-66 ºC	Podoviridae	BetaProteobacteria	4.2	RNA-66 ºC	Myoviridae	Betaproteobacteria	2.5
RNA-58 ºC	Siphoviridae	Verrucomicrobia	0.1	RNA-66 ºC	Podoviridae	Enviromental	0.6	RNA-66 ºC	Myoviridae	Alphaproteobacteria	3.5
RNA-58 ºC	Siphoviridae	Methanobacteria	0.2	--	--	--	--	--	--	--	--
RNA-58 ºC	Siphoviridae	Clostridia	0	--	--	--	--	--	--	--	--
RNA-58 ºC	Siphoviridae	BetaProteobacteria	0	--	--	--	--	--	--	--	--
RNA-66 ºC	Siphoviridae	Actinobacteria	43.9	--	--	--	--	--	--	--	--
RNA-66 ºC	Siphoviridae	Bacilli	28.3	--	--	--	--	--	--	--	--

RNA-66 ºC	Siphoviridae	Gammaproteobacteria	24.1	--	--	--	--	--	--	--	--
RNA-66 ºC	Siphoviridae	Flavobacteriia	1.3	--	--	--	--	--	--	--	--
RNA-66 ºC	Siphoviridae	Alphaproteobacteria	0.4	--	--	--	--	--	--	--	--
RNA-66 ºC	Siphoviridae	Verrucomicrobia	0	--	--	--	--	--	--	--	--
RNA-66 ºC	Siphoviridae	Methanobacteria	0.2	--	--	--	--	--	--	--	--
RNA-66 ºC	Siphoviridae	Clostridia	1.7	--	--	--	--	--	--	--	--
RNA-66 ºC	Siphoviridae	BetaProteobacteria	0.1	--	--	--	--	--	--	--	--

Supplementary Table S4. Relative abundance and transcriptomic expression (RPKM) of *Mastigocladus* sp. CRISPR *loci* and TC-CHP58. Reads were normalized by size and depth of sequencing. Abundance and expression of *Mastigocladus* RUBISCO was used as reference of the cyanobacterial presence and metabolic activity.

Sample T ºC	RUBISCO-DNA-RPKM	DNA_TC-CP58-RPKM	RUBISCO-RNA-RPKM	CRISPR-RNA-RPKM	RNA_TC-CHP58-RPKM
48	36.31	39.58	315.46	413.34	3.79
58	74.86	19.40	397.37	427.61	8.24
66	21.55	155.47	2.26	3.47	0.28

Supplementary Table S5. Number of Single nucleotide variants in TC-CHP58 ORFs at different metagenomes of Porcelana temperature gradient.

ORFs	Start	End	48_SNVs	58_SNVs	66_SNVs	Length
ORF_1-DNA polymerase	40528	1638	36	23	17	1850
ORF_2-Hypothetical protein	1619	1909	11	4	3	290
ORF_3-DNA primase/helicase	1960	4470	129	79	50	2510
ORF_4-Hypothetical protein	4553	5041	39	15	27	488
ORF_5-Hypothetical protein	5038	5220	12	2	2	182
ORF_6-Hypothetical protein	5844	6023	12	4	2	179
ORF_7-dTMP kinase	6246	6839	36	22	14	593
ORF_8-Hypothetical protein	6960	7211	12	12	4	251
ORF_9-Hypothetical protein	7589	7987	48	37	29	398
ORF_10-Hypothetical protein	8158	8556	61	35	38	398
ORF_11-Hypothetical protein	11435	11704	7	1	0	269
ORF_12-Hypothetical protein	11722	12273	18	16	4	551
ORF_13-Hypothetical protein	12292	12780	19	20	11	488
ORF_14-Hypothetical protein	12820	13173	12	5	2	353
ORF_15-Terminase	13177	14886	58	18	13	1709
ORF_16-Portal protein	14886	16931	65	41	34	2045
ORF_17-Scaffold protein	16928	17842	26	9	8	914
ORF_18-Major capsid protein	17862	18896	28	18	14	1034
ORF_19-Tail tubular protein A	18977	19198	8	1	1	221
ORF_20-Tail tubular protein B	19195	19887	19	5	2	692
ORF_21-Hypothetical protein	19889	23071	85	38	21	3182
ORF_22-Hypothetical protein	23088	23357	4	2	0	269
ORF_23-Hypothetical protein	23360	24559	33	17	10	1199
ORF_24-Internal protein/peptidase	24552	27917	104	62	23	3365
ORF_25-Hypothetical protein	27919	31536	78	35	25	3617
ORF_26-Tail protein	31542	34883	94	58	25	3341
ORF_27-Hypothetical protein	34909	35472	22	15	7	563
ORF_28-Hypothetical protein	35605	35790	3	3	0	185
ORF_29-Hypothetical protein	35848	36204	13	7	2	356
ORF_30-Tail Fiber	36245	36679	25	14	7	434

ORF_31-Hypothetical protein	36642	36806	6	5	3	164
ORF_32-N-acetylmuramoyl-L-alanine amidase	36884	37444	19	12	6	560
ORF_33-Hypothetical protein	37428	37634	11	8	5	206
ORF_34-Hypothetical protein	37637	37828	13	9	7	191
ORF_35-Deoxycytidine triphosphate deaminase	37835	38419	13	8	3	584
ORF_36-Hypothetical protein	38434	38697	11	8	7	263
ORF_37-Hypothetical protein	38694	39542	36	19	20	848
ORF_38-Hypothetical protein	39611	39880	9	11	7	269
ORF_39-Hypothetical protein	39892	40182	12	8	7	290
